# Gambling disorder gender analysis: social strain, gender norms, and self-control as risk factors

**DOI:** 10.3389/fsoc.2024.1436066

**Published:** 2024-09-20

**Authors:** Pui Kwan Man

**Affiliations:** Department of Sociology, Hong Kong Shue Yan University, North Point, Hong Kong SAR, China

**Keywords:** gambling disorder, gender, strain, gender norms, self-control, Chinese married couples

## Abstract

**Introduction:**

Gender differences in problem gambling have attracted much attention in recent gambling literature. However, relatively little is known about how gender norms relate to social strain and self-control in predicting gambling disorder within a spousal context. This study aimed to increase knowledge about gambling disorder in Chinese married couples by assessing the three-way interaction effects between social strain, self-control, and gender norms.

**Methods:**

A total of 1,620 Chinese married couples were recruited from a representative sample of households in Hong Kong.

**Results:**

The results of the generalized ordered logit model revealed the self-control mitigation effect of composite strain on the propensity for gambling disorder is strong in men who accept traditional gender norms. In contrast, in women who accept traditional gender roles, self-control attenuates the effect of recent stressful life events on the propensity for gambling disorder, but self-control exacerbates the effect of negative relationships with offspring on the propensity for gambling disorder.

**Discussion:**

Although reinforcing self-control is a protective factor that can alleviate social strain and disordered gambling for both men and women, the prominent contribution of gender norms to the self-control exacerbation effect deserves close attention for social workers who provide services to these gambling families.

## 1 Introduction

Approximately 40 to 80% of the global population participates in gambling activities. Studies report that gambling prevalence in Australia, the U.K., and the U.S.A. is 39, 48, and 78%, respectively; with pathological gambling ranging from 0.7 to 1.1% (Armstrong and Carroll, 2017; Gambling Commission, 2017; Welte et al., 2015). Similarly, gambling participation has increased in Asian populations; the gambling prevalence in Macau, Singapore, and Hong Kong is 51.5, 52, and 61.5%, respectively, with pathological gambling ranging from 0.9 to 2.5% [Hong Kong Polytechnic University (HKPU), 2017; Institute for the Study of Commercial Gaming (ISCG), 2016; National Council of Problem Gambling (NCPB), 2018].

In both Asia and the West, most problem gamblers are male; they typically rely on welfare, have low education levels, and low household income. These are identified as global factors in gambling (Armstrong and Carroll, 2017; Gambling Commission, 2017; Hong Kong Polytechnic University (HKPU), 2017; National Council of Problem Gambling (NCPB), 2018). However, gambling problems have gradually increased in women over the past three decades (Brown and Coventry, 1997; Holdsworth et al., 2012; Tang et al., 2007). In Hong Kong, Macau, and Singapore 47, 50, and 52%, respectively, of the problem gambling population are married individuals. This suggests a higher risk among married people of developing pathological gambling than their unmarried counterparts [Hong Kong Polytechnic University (HKPU), 2017; National Council of Problem Gambling (NCPB), 2018; Tung Wah Group of Hospitals, 2019].

There is a growing focus on addressing gender disparities in gambling literature. For example, male gamblers are often driven by financial incentives, while female gamblers are more motivated by social factors like everyday challenges, marital discord, and feelings of boredom and isolation associated with an “empty nest,” rather than purely financial gain (Brown and Coventry, 1997; Holdsworth et al., 2012; Tang et al., 2007; Wong et al., 2013). Over the past three decades, a segment of the gambling literature has emphasized social strain as a predictor of problem gambling for both genders. Roberts et al. (2017) highlighted that both childhood and recent stressful life events were predictive of disordered gambling among male gamblers. However, the impact of strain stemming from specific social spheres—such as family, work, and peers—on men’s problem gambling remains unclear. In contrast, studies on gambling have identified various sources of social strain for female gamblers, including negative childhood experiences (Boughton and Falenchuk, 2007; Cheung, 2015), stress related to family caregiving roles (Lesieur and Blume, 1991), strained relationships with peers (Trevorrow and Moore, 1998), and experiences of intimate partner violence (Korman et al., 2008). Yet, no prior study has investigated gender variances among married couples within the overall gambling population. Earlier research has not been able to directly compare the influences of different social strain domains on disordered gambling in the context of spousal relationships. Therefore, it is crucial to develop an initial comprehension of potential gender differences in gambling behaviors among married couples.

Moreover, existing literature underscores the significance of gender role socialization in elucidating gender-related aspects of gambling behaviors, which are fundamentally shaped by gender norms. However, empirical examinations of gender norms within the realm of gambling remain scarce in current literature. Hence, the primary objective of this study is to explore the significance of gender norms concerning social strain and self-control concurrently. The subsequent sections will delve into elucidating the relationships among these factors.

## 2 Literature review

### 2.1 Theoretical perspectives on gambling problems

Gambling research underscores the influence of gender role socialization on gender differences in gambling motivations, gambling preferences, and gambling behaviors. Men are often socialized to embody traits such as independence, aggression, and competitiveness. Society tends to associate masculinity with acts of skill and courage. High-stakes gambling, in particular, may provide men with an avenue to showcase their skillfulness, fearlessness, and desire for competition and risk-taking. As a result, men are more likely to engage in competitive and strategic gambling activities, such as sports betting and card games, which align with traditional notions of masculinity. In contrast, women are often socialized to be passive and dependent, and they may gravitate towards luck-based and non-strategic forms of gambling. Activities such as lotteries, bingo, electronic gaming machines, and video poker, which require fewer skills, tend to be more appealing to women. For women, gambling can serve as a means of escape or an emotion-based coping mechanism (Brown and Coventry, 1997; Holdsworth et al., 2012; Wong et al., 2013). Gender socialization is governed by gender norms, which provide guidelines for men and women to develop their gender identities. However, individuals exhibit different levels of gender norm acceptance. Surprisingly, empirical testing of the effects of gender norms on problem gambling is rare. This study fills the research gap by linking two theoretical perspectives to empirically examine gender norms in gambling disorder.

Agnew’s (1992) general strain theory (GST) defines strain as negative relationships with people, and categorizes strain into three broad types: failure to achieve positively valued goals, loss of positively valued stimuli, and confrontation with negative stimuli. These strain categories have a cumulative effect on deviance propensity. Strain may produce negative emotions such as anger and depression, which impose pressure to take corrective action. Crime and deviance are possible responses. GST also identifies gender differences in the experience of strain. Owing to gender role socialization, men are concerned with financial or status-related strain, whereas women are vulnerable to relationship-based strain (Broidy and Agnew, 1997). These propositions support the importance of gender role socialization (Broidy and Agnew, 1997; De Coster, 2005; Keith et al., 2015). In essence, gender norms play a crucial role in the development of gender role socialization. These norms provide guidelines for individuals, prescribing acceptable boundaries of behavior for women and men that align with the gender division of labor and male power (Seguino, 2007). Gender socialization occurs based on these norms, shaping individuals’ gender identity. It is important to note that individuals’ adherence to traditional masculine or feminine traits varies, influenced by their acceptance of gender norms. While the significance of gender norms is widely acknowledged in GST studies, empirical examination of this relationship is still lacking. A key contribution of this study is its explicit focus on the level of acceptance of traditional gender norms and its impact on the gender-deviance nexus. Specifically, the study aims to assess the extent to which individuals, both men and women, who conform to traditional gender norms are likely to exhibit gambling disorder in response to social strain. GST is gradually being applied to gambling problems research (Cheung, 2015, 2016; Eitle and Taylor, 2010; Greco and Curci, 2017; Man and Cheung, 2022). Eitle and Taylor (2010) found that acute stressful events influence gambling behavior than chronic strain. Anger has also been positively associated with pathological gambling. Greco and Curci (2017) found positive effects of strain on gambling, but did not confirm the role of depressive emotions. Cheung (2016) linked GST with self-control theory to explain pathological gambling in Chinese adolescents. Although social strain and low self-control independently predict pathological gambling, high self-control can reduce the effects of social strain on pathological gambling. In other words, high self-control acts as a safeguard against the impact of social strain on problem gambling.

Gottfredson and Hirschi’s (1990) self-control theory asserts that individuals with low self-control are prone to deviance and *vice-versa*. Low self-control is theorized as a lifetime construct that is fairly fixed by ages 8 to 10 and stems from insufficient parental socialization during childhood. People with low self-control tend to exhibit six characteristics: impulsivity, risk-seeking behavior, a preference for easy/simple tasks, a preference for physical rather than mental activities, self-centered orientation, and a volatile temper. Gottfredson and Hirschi (1990) suggest that men have lower self-control than women, and therefore have a higher likelihood of deviance. Self-control theorists argue that this gender disparity results from parenting practices and gender role socialization. Parents must “monitor the child’s behavior, actually practice surveillance, recognize deviant behavior when it occurs, and punish or disapprove such behavior” to increase their children’s self-control (Gottfredson and Hirschi, 1990, p. 97). Per traditional gender norms, the male gender role emphasizes competitive, assertive, and aggressive behaviors, whereas the female role emphasizes submissive, passive, and caring behaviors. Parents then follow these societal expectations for gender-appropriate conduct and are more likely to correct their daughters’ misbehavior than their sons’. Consequently, daughters are expected to develop higher self-control than sons. In recent decades, scholars in the field of deviance studies have dedicated further efforts to examine self-control as a gender-specific factor that conditions social strain. Cheung and Cheung (2010) demonstrated how self-control theory provides valuable insights into the relationship between gender, strain, and delinquency. They found that self-control mitigates the impact of strain on delinquency among female adolescents but not male adolescents. The study also revealed that the effect of strain on delinquency is more pronounced in males than females, while self-control does not alleviate stress for males. Interestingly, among males, exposure to coercive parenting tends to decrease delinquent behavior when combined with low self-control. These findings partially explain the gender gap observed in deviant behavior by highlighting the gender disparity in the moderating effect of self-control on strain. This present study, which contributes to the limited empirical research on self-control theory in the Chinese context, builds upon the existing literature and extends the application of self-control theory to investigate the role of self-control as a gender-specific conditioning factor in relation to another form of deviant behavior: gambling disorder. Self-control theory suggests that gambling is a crime-analogous act that satisfies the same basic urges that facilitate criminal behavior (Barnes et al., 2005; Cheung, 2016). Therefore, it is theoretically sound to posit that those with low self-control may easily indulge in gambling. Although research has identified deficient self-control as a major factor in problem gambling and high self-control can ameliorate the effect of social strain on problem gambling (Barnes et al., 2005; Bergen et al., 2012; Cheung, 2016; Jeong et al., 2020), the question of whether self-control is a gender-specific conditioning factor has not been empirically examined.

The theoretical framework of our study posits that the interplay between social strain, self-control, and gender norms is pivotal in understanding the propensity for gambling disorder by gender. While previous research has predominantly focused on the individual impacts of these factors, the complex dynamics of their three-way interactions remain underexplored. By delving into this intricate relationship, I aim to provide a more nuanced understanding of how social strain, self-control, and adherence to gender norms collectively shape individuals’ vulnerability to gambling disorder. Based on GST and self-control theory, high self-control is a protector against social strain in problem gambling (Cheung, 2016). Recently, Man and Cheung (2022) found that gender norms increase social strain’s effect on gambling disorder in men but decrease it in women. This study builds on the previous literature and further connects self-control theory to test the three-way interaction effects of social strain, self-control, and gender norms on the propensity for gambling disorder within a Chinese spousal context. According to self-control theory, females are believed to exhibit higher levels of self-control than males due to gender socialization, which is guided by prevailing gender norms. This study postulates that high self-control can alleviate the impact of social strain on the propensity for gambling disorder. Given that females are typically encouraged to cultivate feminine attributes and are closely supervised by parents, their enhanced self-control might mitigate the effects of social strain on their susceptibility to gambling disorders. This mitigation effect on self-control could be more pronounced in females who adhere to traditional gender norms compared to those who do not. Conversely, males who conform to traditional gender norms may display weaker self-control than their non-conforming counterparts. Consequently, the mitigation effect of self-control could be less pronounced in males who adhere to traditional gender norms than in those who do not. The following hypotheses are structured to address the unique aspects of this complex interaction, enabling us to go beyond the traditional two-way relationships identified in the existing literature.

H1: The self-control mitigation effect of social strain on the propensity for gambling disorder is weaker in traditional men than in non-traditional men.

H2: The self-control mitigation effect of social strain on the propensity for gambling disorder is stronger in traditional women than in non-traditional women.

## 3 Materials and methods

### 3.1 Sample

The study data were from the project “Social Control, Strain and Couple Dynamics Affecting Gender Disparities in Gambling: A Study of Married Couples in Hong Kong” (General Research Fund Project No. 442410), which was conducted from December 2010 to May 2013. It involved a territory-wide cross-sectional survey of 1,620 married Chinese couples. This study selected a stratified random-cluster representative sample of households located in New Territories, Kowloon, and Hong Kong Island. Public and private housing were identified in each stratum, and 270 households were then selected for each type. The sample comprised both husband and wife in each household; participants were between 21 and 50 years old and were recruited for face-to-face interviews. Their average educational attainment was upper secondary/university matriculation level. Husbands were primarily manual workers, and most wives were clerks/service workers. Most married Chinese couples have children and live in public housing. We used an anonymous standardized questionnaire in the interviews, and all items were presented in Chinese. To ensure data confidentiality, respondents were interviewed at their homes, independent of their spouses. If a spouse’s absence could not be guaranteed, the interview was conducted at a venue outside the home.

### 3.2 Measures

#### 3.2.1 Gambling disorder

The dependent variable was gambling disorder, with measurement based on the DSM-V diagnostic instrument comprising nine “yes” or “no” items assessing nine common symptoms of gambling disorder: loss of control, tolerance, withdrawal, chasing losses, preoccupation with gambling, use of gambling to escape reality, lying, risking one’s education/job/relationship, and financial difficulties that require bailouts from others (American Psychiatric Association, 2013). The Cronbach’s α was 0.9, indicating a high degree of reliability. We identified three levels of gambling disorder in our analysis: non-gambler, no/low-risk disordered gamblers (no or one affirmative item), and at-risk/probable disordered gamblers (two or more affirmative items).

#### 3.2.2 Social strain variables

Eight social strain variables operationalized the three forms of social strain. Regarding failure to achieve job goals, respondents reported the extent in the preceding 2 years they had troubles with boredom, income instability, stress at work, unfair treatment at work, a gap between expected and actual returns from work, and failure to get the job they wanted (1 = never to 4 = always; six-item scale, α = 0.85). Failure to achieve household well-being was measured by how often in the preceding 2 years respondents had been troubled by household fatigue, excessive household work, and a lack of personal space in the living environment (1 = never to 4 = always; three-item scale, *α* = 0.76). For recent stressful life events, respondents listed the stressful life events that they experienced and felt sad about over the preceding 2 years. The variable of recent stressful life events was a count measure that included divorce, unemployment, demotion, accident or serious illness, death of a parent, reliance on public assistance, public assistance suspension, and financial problems. The eight items were coded as 0 or 1 and then summed to create an index for recent stressful life events. Another count measure was childhood/adolescent stressful life events. Childhood/adolescent stressful life events (before age 18) included parental separation, death of a parent, school dropout, impoverished living environment, living involuntarily with a foster family, failure to attain university admission, serious illness experienced by a parent, and the sudden death of relatives or friends. These eight items were also coded as 0 or 1 and summed to form an index. It is important to note that stressful life events serve as causal indicators rather than effect indicators of social strain. Each life event represents a determinant of an individual’s exposure to strain rather than a consequence of it. While effect indicators for the same latent variable should be positively associated, this is not necessarily true for causal indicators (Bollen and Ting, 2000; Cheung and Cheung, 2010; Cheung, 2015). These causal indicators of stressful life events have been widely used in research on GST. For negative relationships with offspring, the questions involved: how often they encountered stress or estrangement from their children, and whether they had conflicts with their children over the preceding 2 years (1 = never to 4 = always; two-item scale, α = 0.71). For negative relationships with peers, respondents reported how often in the preceding 2 years they had conflicts with peers or had failed to return money owed to peers (1 = never to 4 = always, two-item scale, *α* = 0.70). Negative relationships with colleagues were measured by asking to what extent in the preceding 2 years respondents were under stress because of their poor relationship with managers, conflicts with workmates, or isolation from colleagues (1 = never to 4 = always, three-item scale, *α* = 0.82). Marital conflict was assessed by asking to what extent in the preceding 2 years respondents had experienced stress because of communication problems with their spouse, arguments over financial issues, a lack of consideration of thoughts and feelings, or severe marital conflicts (1 = never to 4 = always, four-item scale, *α* = 0.89). To estimate the cumulative effects of social strain on gambling disorder propensity, I aggregated the eight social strain predictors into composite strain, with higher scores indicating higher levels of composite strain.

#### 3.2.3 Negative emotions

The negative emotions addressed in this study were anger and depression. Respondents reported to what extent in the preceding 2 years they had felt angry or depressed when they encountered difficulties (1 = never to 4 = always). A two-year timeframe was used to capture the influence of respondents’ negative affect on gambling disorder likelihood. Higher scores reflect higher levels of anger and depression.

#### 3.2.4 Gender norms

The gender norms variable represents the gender attributes concept, which may be reflected in individuals’ perceptions of the primacy of their breadwinner role, perceptions of femininity, male privilege, attitudes toward separate gendered spheres, household utility, and the effect of women’s work on relationship quality (Davis and Greenstein, 2009). Respondents reported their agreement with statements concerning traditional gender role expectations (1 = strongly disagree to 4 = strongly agree; twelve-item scale, *α* = 0.72). Item responses were summed to form a composite score ranging from 12 to 48. Higher scores reflect a greater acceptance of traditional gender norms.

#### 3.2.5 Self-control

I used the 23-item scale introduced by Grasmick et al. (1993) to measure self-control. This instrument is the most widely used in deviance research and has been demonstrated to have good construct reliability in both Eastern and Western cultures (Cheung, 2016; Romero et al., 2003; Vazsonyi et al., 2004). The scale encompasses Gottfredson and Hirschi’s (1990) six elements: impulsivity, risk-taking, self-centeredness, a preference for simple over complicated tasks, a preference for physical over mental activities, and a volatile temper. The items are rated on a four-point scale (1 = strongly agree to 4 = strongly disagree, *α* = 0.90), with lower scores indicating lower self-control.

#### 3.2.6 Control variables: social bonds

I have considered the potential impacts of social bonds and social learning, which involve mechanisms other than social strain, in relation to gambling disorder. The social bonding variables are presumed to exert informal social control that acts as a protective factor against gambling problems. On the other hand, the social learning variables capture the acquisition of pro-gambling behaviors and attitudes that contribute to gambling problems. Additionally, I have included socio-demographic variables as control variables in the analysis.

Social bonding theorists assert that individuals who have strong bonds with significant others are less prone to deviance because they do not want to harm their affection ties (Cheung, 2015, 2016; Kalischuk et al., 2006). Therefore, attachment to conventional others may impose informal control over deviant behaviors, such as pathological gambling. I operationalized social bonds through two variables: family support and attachment to one’s spouse. I used an eight-item scale to measure these two variables (1 = strongly disagree to 4 = strongly agree, α = 0.85). The two variables were summed, and lower scores were indicative of weaker social bonds.

#### 3.2.7 Control variables: social learning

Social learning theorists assert that gambling is a learned behavior. Gambling spouses and peers may inculcate pro-gambling behaviors and beliefs in individuals (Cheung, 2015, 2016; Langhinrichsen-Rohling et al., 2004). I used a pro-gambling social learning variable that included gambling spouse or peer measures. Respondents were asked how often their spouses gambled (1 = never to 4 = always; four-item scale), and how many of their friends had gambling habits (1 = none to 4 = many); I summed the two scores to form this variable.

#### 3.2.8 Control variables: socio-demographics

Control variables included age, educational attainment, and occupational status. Age was a continuous measure of years. Educational attainment was “below lower-secondary level” (scored as 1), “lower-secondary level” (scored as 2), “upper secondary level/university matriculation” (scored as 3), “sub-degree” (scored as 4), or “Bachelor’s degree or above” (scored as 5). Occupational status was assessed by respondents’” self-described occupation, “unemployed/housewife/househusband” (scored as 1), “manual worker” (scored as 2), “clerk/service worker” (scored as 3), or “manager/administrator/professional/self-employed” (scored as 4).

A small number of observations were missing for each of the independent variables, ranging from 0.1 to 2.6% of cases. To address these missing values, I employed a multiple imputation method using the Amelia program (King et al., 2001) to replace the missing values.

### 3.3 Statistical analysis

Initially, gambling severity by gender and descriptive statistics were presented for all variables of interest. T-tests were conducted to compare the means of independent variables between men and women, aiming to determine statistically significant differences in means across gender groups. Subsequently, a series of multivariate models were employed to explore the relationships among social strain, gender norms, self-control, and the propensity for gambling disorder. These models assessed the main effects, two-way, and three-way interaction effects of predictor variables on gambling disorder propensity separately for men and women.

For the analysis, three comparison groups were created: non-gamblers, no/low-risk disordered gamblers, and at-risk/probable disordered gamblers. Considering the ordinal nature of the dependent variable, an ordered response model was deemed appropriate. Ordinal logit models are commonly used in cases involving ordinal dependent variables. However, one limitation of the ordered logit model is the assumption of equal estimated parameters for each independent variable, known as the parallel lines or proportional odds assumption, which is often violated. To address this, a test of the proportional odds assumption was conducted. It was found that the assumption did not hold for several covariates, namely negative relationships with offspring, marital conflict, gender norms, self-control, and age. To account for this violation, the model was re-estimated using the Generalized Ordered Logit (GOL) model, as discussed by Williams (2006, 2016). The GOL model allows for the relaxation of the parallel lines assumption. In the present study, there are three categories: the highest ranked outcome is at-risk/probable disordered gamblers (coded as 3), followed by no/low-risk disordered gamblers (coded as 2), and finally non-gambler as the lowest ranked outcome (coded as 1). The GOL model in the current study was estimated using the estimator “gologit2” written for Stata. The independent variables were standardized as z-scores in all multivariate analyses to reduce multicollinearity. All statistical analyses were performed using Stata, with statistical significance defined as alpha <0.05.

## 4 Results

Table 1 displays the DSM-V diagnosed gambling disorder levels for our sample. Gambling disorder was more pronounced in men than women; only 26.8% of men were non-gamblers. Nearly 64.5% of men fell within the range of no/low-risk disordered gamblers (DSM-V = 0–1), whereas only 47.5% of women fell in the same range. A higher proportion of men (8.7%) than women (1.9%) were at-risk/probable disordered gamblers (DSM-V = 2–9). The proportion of male gamblers surpasses that of female gamblers across all levels. A chi-square test indicated a significant gender difference in gambling disorder, where men showed more severe levels of gambling than women.

**Table 1:** Gambling disorder levels by gender.

Gambling disorder levels	Men (*N* = 1,620)	Women (*N* = 1,620)
Non-gambler	26.8% (*n* = 434)	50.6% (*n* = 820)
No/low-risk disordered gamblers (DSM-V = 0–1)	64.5% (*n* = 1,045)	47.5% (*n* = 769)
At-risk/probable disordered gamblers (DSM-V = 2–9)	8.7% (*n* = 141)	1.9% (*n* = 31)

Chi-square test with 2 degrees of freedom = 231.159, *p* < 0.001.

Table 2 presents descriptive statistics for all independent variables stratified by gender, along with the t-test comparing men and women at the mean levels. The results revealed significant differences in six out of the nine social strain variables between men and women. Specifically, men reported significantly higher levels of failure to achieve job goals, recent stressful life events, negative relationships with peers, and negative relationships with colleagues. However, women experienced more strain as a result of marital conflict and their failure to achieve household well-being. On the other hand, there were no significant differences in the mean scores for childhood/adolescent stressful life events, negative relationships with offspring, and composite strain between men and women. Notably, t-test results indicated significantly higher levels of anger and depression among women compared to men. Conversely, men exhibited more traditional gender attitudes than women. As expected, men had lower levels of self-control compared to women. In terms of social bonds, there were no significant differences in mean scores between men and women. However, men had significantly higher pro-gambling social learning compared to women. Regarding socio-demographics, women were significantly younger than men, while there were no significant differences in mean scores for educational attainment and occupational status based on gender.

**Table 2:** Descriptive statistics for variables by gender.

Variable	Range Min Max		Full sample Mean SD		Male sample Mean SD		Female sample Mean SD		*T*-test statistics	
Social strain										
Failure to achieve job goals	1	24	11.30	4.26	11.70	4.58	10.89	3.87	5.406	***
Failure to achieve household well-being	1	12	5.23	2.43	4.70	2.09	5.75	2.62	−12.676	***
Childhood/adolescent stressful life events	0	8	1.45	1.36	1.48	1.41	1.42	1.32	1.236	n.s.
Recent stressful life events	0	8	0.76	1.13	0.83	1.20	0.69	1.05	3.475	***
Negative relationships with offspring	1	8	2.79	1.18	2.77	1.20	2.80	1.16	−0.758	n.s.
Negative relationships with peers	1	8	2.64	1.00	2.76	1.08	2.52	0.89	6.834	***
Negative relationships with colleagues	1	12	4.22	1.61	4.29	1.70	4.14	1.50	2.798	**
Marital conflict	1	16	6.69	3.00	6.55	2.89	6.83	3.11	−2.659	**
Composite strain	20	77	35.05	10.88	35.07	11.45	35.04	10.29	0.081	n.s.
Negative emotions										
Anger	1	4	1.91	0.86	1.87	0.84	1.95	0.88	−2.407	*
Depression	1	4	1.82	0.86	1.78	0.83	1.85	0.88	−2.362	*
Gender norms	1	48	28.29	4.70	29.29	4.79	27.29	4.38	12.414	***
Self-Control	1	92	67.69	8.42	67.03	8.59	68.35	8.19	−4.458	***
Social bonds	1	32	26.55	3.63	26.46	3.61	26.64	3.66	0.081	n.s.
Social learning	1	8	4.08	1.29	4.17	0.03	3.99	0.03	4.028	***
Socio-demographics										
Age (in years)	21	50	40.23	6.86	42.10	6.55	38.37	6.66	16.091	***
Educational attainment	1	5	2.61	0.99	2.63	1.01	2.59	0.98	1.226	n.s.
Occupational status	1	4	2.32	0.88	2.29	0.78	2.35	0.97	−1.686	n.s.

*T*-tests assess the mean differences of independent variables between men and women.

*p < 0.05, **p < 0.01, ***p < 0.001, n.s.: Not significant.

Table 3 shows the estimated results of the effects of all independent variables on men’s gambling disorder propensity using the GOL model. Models 1 and 2 display the main effects of all variables, while Models 3 to 6 demonstrate the two-way and three-way interaction effects. Regarding the main effects, nine of the regressors were statistically significant across their respective categories. The variables that increased the odds of gambling severity were childhood/adolescent stressful life events, negative relationships with offspring, negative relationships with peers, marital conflict, composite strain, and social learning. Conversely, the coefficients of self-control, educational attainment, and occupational status were negative and statistically significant, indicating that men with high self-control, higher education levels, and greater occupational prestige tended to have a lower likelihood of being indulged in gambling disorder.

**Table 3:** Results of social strain, self-control, and gender norms on gambling disorder propensity in men using the GOL model.

	Model 1		Model 2		Model 3		Model 4		Model 5		Model 6	
	Main effects				Two-way interaction effects				Three-way interaction effects			
Variables	C1	C2	C1	C2	C1	C2	C1	C2	C1	C2	C1	C2
Social strain												
Failure to achieve job goals	0.133	0.133			0.116	0.116			0.113	0.113		
	(0.075)	(0.075)			(0.076)	(0.076)			(0.078)	(0.078)		
Failure to achieve household well-being	−0.095	−0.095			−0.108	−0.108			−0.123	−0.123		
	(0.067)	(0.067)			(0.070)	(0.070)			(0.072)	(0.072)		
Childhood/adolescent stressful life events	0.069	0.312***			0.091	0.091			0.100	0.100		
	(0.065)	(0.094)			(0.062)	(0.062)			(0.063)	(0.063)		
Recent stressful life events	−0.042	−0.042			−0.048	0.158			−0.058	0.139		
	(0.062)	(0.062)			(0.073)	(0.092)			(0.073)	(0.094)		
Negative relationships with offspring	−0.037	0.187*			−0.058	0.151			−0.063	0.246*		
	(0.072)	(0.088)			(0.075)	(0.097)			(0.078)	(0.115)		
Negative relationships with peers	0.150*	0.150*			0.158*	0.158*			0.144*	0.144*		
	(0.066)	(0.066)			(0.070)	(0.070)			(0.073)	(0.073)		
Negative relationships with colleagues	−0.007	−0.007			0.023	0.023			0.050	0.050		
	(0.069)	(0.069)			(0.073)	(0.073)			(0.076)	(0.076)		
Marital conflict	−0.072	0.478***			−0.047	0.446***			−0.079	0.365**		
	(0.086)	(0.111)			(0.089)	(0.119)			(0.090)	(0.131)		
Composite strain			0.060	0.709***			0.070	0.732***			0.058	0.653***
			(0.077)	(0.103)			(0.078)	(0.107)			(0.080)	(0.121)
Negative emotions												
Depression	−0.078	−0.078	−0.084	−0.084	−0.075	−0.075	−0.084	−0.084	−0.073	−0.073	−0.087	−0.087
	(0.072)	(0.072)	(0.071)	(0.071)	(0.073)	(0.073)	(0.072)	(0.072)	(0.074)	(0.074)	(0.072)	(0.072)
Anger	0.029	0.029	0.035	0.035	0.037	0.037	0.036	0.036	0.032	0.032	0.033	0.033
	(0.073)	(0.073)	(0.072)	(0.072)	(0.074)	(0.074)	(0.072)	(0.072)	(0.075)	(0.075)	(0.072)	(0.072)
Gender norms (GN)	0.021	0.021	−0.030	0.207	0.027	0.027	−0.029	0.207	−0.084	0.285*	−0.088	0.261*
	(0.056)	(0.056)	(0.061)	(0.106)	(0.056)	(0.056)	(0.061)	(0.106)	(0.068)	(0.120)	(0.067)	(0.119)
Self-control (SC)	−0.164*	−0.495***	−0.165**	−0.477***	−0.162*	−0.481***	−0.158*	−0.497***	−0.189**	−0.189**	−0.135*	−0.403**
	(0.064)	(0.112)	(0.064)	(0.113)	(0.067)	(0.118)	(0.065)	(0.116)	(0.065)	(0.065)	(0.066)	(0.127)
Social bonds	−0.072	−0.072	−0.080	−0.080	−0.078	−0.078	−0.080	−0.080	−0.077	−0.077	−0.076	−0.076
	(0.061)	(0.061)	(0.058)	(0.058)	(0.061)	(0.061)	(0.058)	(0.058)	(0.062)	(0.062)	(0.059)	(0.059)
Social learning	0.422***	0.422***	0.432***	0.432***	0.423***	0.423***	0.430***	0.430***	0.431***	0.431***	0.433***	0.433***
	(0.058)	(0.058)	(0.058)	(0.058)	(0.059)	(0.059)	(0.058)	(0.058)	(0.060)	(0.060)	(0.058)	(0.058)
Socio-demographics												
Age	0.893	0.893	0.742	0.742	0.813	0.813	0.732	0.732	0.885	0.885	0.757	0.757
	(0.633)	(0.633)	(0.629)	(0.629)	(0.639)	(0.639)	(0.630)	(0.630)	(0.642)	(0.642)	(0.631)	(0.631)
Age squared	−0.002	−0.002	−0.001	−0.001	−0.001	−0.001	−0.001	−0.001	−0.002	−0.002	−0.001	−0.001
	(0.001)	(0.001)	(0.001)	(0.001)	(0.001)	(0.001)	(0.001)	(0.001)	(0.001)	(0.001)	(0.001)	(0.001)
Educational attainment	−0.037	−0.348**	−0.038	−0.337**	−0.050	−0.360**	−0.039	−0.335**	−0.071	−0.359**	−0.048	−0.328**
	(0.064)	(0.113)	(0.063)	(0.112)	(0.064)	(0.114)	(0.063)	(0.112)	(0.065)	(0.116)	(0.063)	(0.112)
Occupational status	−0.113	−0.113	−0.114*	−0.114*	−0.115*	−0.115*	−0.116*	−0.116*	−0.107	−0.107	−0.114*	−0.114*
	(0.058)	(0.058)	(0.057)	(0.057)	(0.059)	(0.059)	(0.057)	(0.057)	(0.059)	(0.059)	(0.058)	(0.058)
Two-way interactions of social strain × SC												
Failure to achieve job goals					−0.125	−0.125			−0.126	−0.126		
					(0.071)	(0.071)			(0.074)	(0.074)		
Failure to achieve household well-being					−0.058	−0.058			−0.030	−0.030		
					(0.066)	(0.066)			(0.072)	(0.072)		
Childhood/adolescent stressful life events					0.019	−0.242**			0.011	−0.254**		
					(0.062)	(0.082)			(0.065)	(0.090)		
Recent stressful life events					0.192**	0.192**			0.190**	0.190**		
					(0.062)	(0.062)			(0.064)	(0.064)		
Negative relationships with offspring					−0.068	−0.068			−0.152	0.088		
					(0.061)	(0.061)			(0.083)	(0.109)		
Negative relationships with peers					0.033	0.033			0.020	0.020		
					(0.065)	(0.065)			(0.070)	(0.070)		
Negative relationships with colleagues					0.098	0.098			0.093	0.093		
					(0.067)	(0.067)			(0.071)	(0.071)		
Marital conflict					0.046	0.046			0.147	−0.259*		
					(0.077)	(0.077)			(0.090)	(0.131)		
Composite strain							0.038	0.038			0.108	−0.133
							(0.053)	(0.053)			(0.061)	(0.106)
Three-way interactions of social strain x SC x GN												
Failure to achieve job goals									−0.009	−0.009		
									(0.069)	(0.069)		
Failure to achieve household well-being									−0.065	−0.065		
									(0.059)	(0.059)		
Childhood/adolescent stressful life events									0.016	0.016		
									(0.056)	(0.056)		
Recent stressful life events									−0.031	−0.031		
									(0.066)	(0.066)		
Negative relationships with offspring									0.070	−0.078		
									(0.061)	(0.067)		
Negative relationships with peers									−0.037	−0.037		
									(0.067)	(0.067)		
Negative relationships with colleagues									0.061	0.061		
									(0.067)	(0.067)		
Marital conflict									−0.161	0.171		
									(0.087)	(0.098)		
Composite strain											−0.132*	0.066
											(0.057)	(0.071)
Constant	3.978	−0.229	3.407	−0.750	3.734	−0.503	3.393	−0.782	3.998	−0.230	3.498	−0.690
	(2.186)	(2.184)	(2.172)	(2.172)	(2.207)	(2.206)	(2.173)	(2.174)	(2.218)	(2.215)	(2.179)	(2.178)
Observations	1,620	1,620	1,620	1,620	1,620	1,620	1,620	1,620	1,620	1,620	1,620	1,620
AIC	2498.056		2505.800		2494.674		2507.286		2499.437		2504.060	
BIC	2632.811		2597.433		2677.940		2604.309		2747.385		2617.253	
LR chi2	299.975		276.231		321.357		276.745		340.594		285.972	
Prob > chi2	0.000		0.000		0.000		0.000		0.000		0.000	
Pseudo R2	0.109		0.101		0.117		0.101		0.124		0.104	

C1: category 1 – non-gambler vs no/low-risk disordered gamblers and At-risk/probable disordered gamblers.

C2: category 2 – non-gambler and no/low-risk disordered gamblers vs At-risk/probable disordered gamblers.

Standard errors in parentheses. ***p < 0.001, **p < 0.01, *p < 0.05.

Models 3 and 4 were conducted to examine whether the effects of social strain variables on gambling disorder propensity were moderated by self-control. After controlling for the main effects of predictors, the results indicated significant and negative interaction effects between self-control and childhood/adolescent stressful life events. Additionally, self-control exhibited positive interaction effects with recent stressful life events. High levels of self-control were found to mitigate the impact of childhood/adolescent stressful life events on gambling severity. However, it was observed that self-control exacerbated the effect of recent stressful life events on men’s gambling severity.

**Figure 1:**
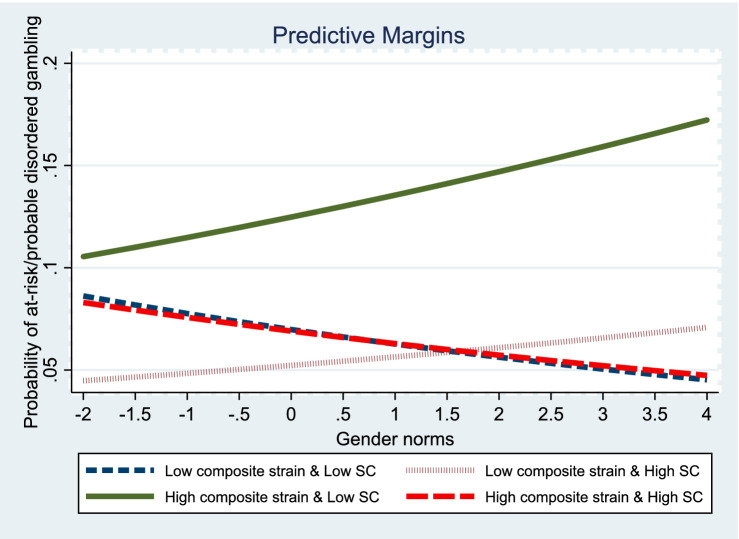
Graph of three-way interaction effects of composite strain, self-control, and gender norms on gambling disorder propensity in men.

Models 5 and 6 further investigate the effects of a three-way interaction (composite strain × self-control × gender norms) on gambling disorder propensity in men. When gender norms were included in the analysis, only one significant interaction effect was observed for men classified as “non-gambler.” Specifically, high self-control mitigated the impact of composite strain on gambling severity in men who adhered to traditional gender norms. This indicates that higher levels of self-control could help traditional men avoid gambling disorder when they encounter composite strain compared to those with lower levels of self-control. To enhance the visualization of the three-way interaction effect, I include a graph in Figure 1.

Table 4 presents the regression results of the effects of all independent variables on gambling disorder propensity in women. Models 1 and 2 display the main effects of all variables, while Models 3 to 6 demonstrate the two-way and three-way interaction effects. Regarding the main effects, childhood/adolescent stressful life events, composite strain, pro-gambling social learning, and greater occupational prestige were found to increase the odds of developing gambling severity in women. On the contrary, failure to achieve household well-being, self-control, and social bonds exhibited negative and statistically significant effects across the categories of “non-gambler” and “no/low-risked disordered gamblers,” suggesting that these three variables serve as protective factors against women’s gambling severity.

**Table 4:** Results of social strain, self-control, and gender norms on gambling disorder propensity in women using the GOL model.

	Model 1		Model 2		Model 3		Model 4		Model 5		Model 6	
	Main effects				Two-way interaction effects				Three-way interaction effects			
Variables	C1	C2	C1	C2	C1	C2	C1	C2	C1	C2	C1	C2
Social strain												
Failure to achieve job goals	0.062	0.062			0.060	0.060			0.079	0.079		
	(0.065)	(0.065)			(0.066)	(0.066)			(0.067)	(0.067)		
Failure to achieve household well-being	−0.229**	0.170			−0.209**	−0.209**			−0.229**	−0.229**		
	(0.073)	(0.187)			(0.072)	(0.072)			(0.074)	(0.074)		
Childhood/adolescent stressful life events	0.171**	0.171**			0.167**	0.167**			0.197***	0.197***		
	(0.057)	(0.057)			(0.058)	(0.058)			(0.060)	(0.060)		
Recent stressful life events	0.004	0.004			−0.028	0.379*			−0.053	0.343		
	(0.062)	(0.062)			(0.065)	(0.190)			(0.067)	(0.192)		
Negative relationships with offspring	0.001	0.001			0.003	0.003			0.021	0.021		
	(0.060)	(0.060)			(0.062)	(0.062)			(0.063)	(0.063)		
Negative relationships with peers	0.024	0.024			0.049	0.049			0.037	0.037		
	(0.062)	(0.062)			(0.065)	(0.065)			(0.067)	(0.067)		
Negative relationships with colleagues	0.075	0.075			0.107	0.107			0.124	0.124		
	(0.063)	(0.063)			(0.065)	(0.065)			(0.066)	(0.066)		
Marital conflict	0.038	0.038			0.043	0.043			0.049	0.049		
	(0.081)	(0.081)			(0.082)	(0.082)			(0.083)	(0.083)		
Composite strain			0.030	0.426*			0.044	0.471**			0.055	0.478**
			(0.069)	(0.167)			(0.069)	(0.167)			(0.070)	(0.167)
Negative emotions												
Depression	−0.023	−0.023	−0.024	−0.024	−0.027	−0.027	−0.025	−0.025	−0.015	−0.015	−0.025	−0.025
	(0.075)	(0.075)	(0.074)	(0.074)	(0.076)	(0.076)	(0.074)	(0.074)	(0.076)	(0.076)	(0.074)	(0.074)
Anger	0.087	0.087	0.075	0.075	0.086	0.086	0.075	0.075	0.075	0.075	0.074	0.074
	(0.076)	(0.076)	(0.075)	(0.075)	(0.076)	(0.076)	(0.075)	(0.075)	(0.077)	(0.077)	(0.075)	(0.075)
Gender norms (GN)	−0.092	−0.092	−0.090	−0.090	−0.094	−0.094	−0.096	−0.096	−0.084	−0.084	−0.077	−0.077
	(0.055)	(0.055)	(0.054)	(0.054)	(0.055)	(0.055)	(0.054)	(0.054)	(0.060)	(0.060)	(0.058)	(0.058)
Self-control (SC)	−0.159**	−0.898***	−0.158**	−0.861***	−0.152*	−1.021***	−0.154**	−0.893***	−0.177**	−1.022***	−0.152*	−0.882***
	(0.060)	(0.228)	(0.059)	(0.235)	(0.061)	(0.247)	(0.060)	(0.232)	(0.062)	(0.244)	(0.060)	(0.231)
Social bonds	0.000	−0.460**	0.013	−0.407*	0.004	−0.469**	0.018	−0.402*	0.008	−0.490**	0.020	−0.401*
	(0.062)	(0.174)	(0.058)	(0.181)	(0.063)	(0.178)	(0.058)	(0.180)	(0.063)	(0.179)	(0.059)	(0.181)
Social learning	0.663***	0.663***	0.655***	0.655***	0.663***	0.663***	0.655***	0.655***	0.660***	0.660***	0.653***	0.653***
	(0.059)	(0.059)	(0.059)	(0.059)	(0.059)	(0.059)	(0.059)	(0.059)	(0.060)	(0.060)	(0.059)	(0.059)
Socio-demographics												
Age	0.811	0.374	0.780	0.348	0.913	0.413	0.772	0.350	0.963	0.445	0.776	0.350
	(0.556)	(0.578)	(0.551)	(0.572)	(0.560)	(0.582)	(0.552)	(0.573)	(0.564)	(0.587)	(0.553)	(0.574)
Age squared	−0.001	−0.001	−0.001	−0.001	−0.002	−0.002	−0.001	−0.001	−0.002	−0.002	−0.001	−0.001
	(0.001)	(0.001)	(0.001)	(0.001)	(0.001)	(0.001)	(0.001)	(0.001)	(0.001)	(0.001)	(0.001)	(0.001)
Educational attainment	−0.012	−0.012	−0.015	−0.015	−0.016	−0.016	−0.022	−0.022	−0.013	−0.013	−0.020	−0.020
	(0.060)	(0.060)	(0.059)	(0.059)	(0.061)	(0.061)	(0.059)	(0.059)	(0.061)	(0.061)	(0.059)	(0.059)
Occupational status	0.145*	0.145*	0.138*	0.138*	0.155**	0.155**	0.142*	0.142*	0.162**	0.162**	0.144*	0.144*
	(0.058)	(0.058)	(0.057)	(0.057)	(0.059)	(0.059)	(0.057)	(0.057)	(0.059)	(0.059)	(0.057)	(0.057)
Two-way interactions of social strain × SC												
Failure to achieve job goals					0.005	0.005			0.023	0.023		
					(0.069)	(0.069)			(0.071)	(0.071)		
Failure to achieve household well-being					−0.003	−0.003			0.000	0.000		
					(0.074)	(0.074)			(0.075)	(0.075)		
Childhood/adolescent stressful life events					0.055	0.055			0.044	0.044		
					(0.060)	(0.060)			(0.061)	(0.061)		
Recent stressful life events					−0.138	0.275			−0.166*	0.258		
					(0.071)	(0.181)			(0.073)	(0.180)		
Negative relationships with offspring					0.136*	−0.253			0.127	−0.243		
					(0.065)	(0.149)			(0.066)	(0.152)		
Negative relationships with peers					0.008	0.008			0.018	0.018		
					(0.067)	(0.067)			(0.068)	(0.068)		
Negative relationships with colleagues					0.117	0.117			0.114	0.114		
					(0.070)	(0.070)			(0.071)	(0.071)		
Marital conflict					−0.006	−0.006			−0.016	−0.016		
					(0.078)	(0.078)			(0.079)	(0.079)		
Composite strain							0.085	0.085			0.091	0.091
							(0.055)	(0.055)			(0.056)	(0.056)
Three-way interactions of social strain x SC x GN												
Failure to achieve job goals									0.106	0.106		
									(0.071)	(0.071)		
Failure to achieve household well-being									0.048	0.048		
									(0.072)	(0.072)		
Childhood/adolescent stressful life events									0.102	0.102		
									(0.058)	(0.058)		
Recent stressful life events									−0.178**	−0.178**		
									(0.065)	(0.065)		
Negative relationships with offspring									0.129*	0.129*		
									(0.062)	(0.062)		
Negative relationships with peers									−0.061	−0.061		
									(0.065)	(0.065)		
Negative relationships with colleagues									0.023	0.023		
									(0.069)	(0.069)		
Marital conflict									−0.031	−0.031		
									(0.078)	(0.078)		
Composite strain											0.050	0.050
											(0.049)	(0.049)
Constant	2.254	−2.775	2.135	−2.921	2.562	−2.572	2.143	−2.945	2.693	−2.465	2.149	−2.933
	(1.664)	(1.683)	(1.649)	(1.671)	(1.674)	(1.694)	(1.653)	(1.675)	(1.687)	(1.706)	(1.655)	(1.677)
Observations	1,620	1,620	1,620	1,620	1,620	1,620	1,620	1,620	1,620	1,620	1,620	1,620
AIC	2288.451		2294.095		2291.988		2293.730		2290.443		2294.711	
BIC	2417.816		2385.728		2475.254		2390.753		2516.831		2397.124	
LR chi2	267.420		247.777		283.883		250.142		301.428		251.161	
Prob > chi2	0.000		0.000		0.000		0.000		0.000		0.000	
Pseudo R2	0.107		0.099		0.113		0.100		0.120		0.100	

C1: category 1 – non-gambler vs no/low-risk disordered gamblers and At-risk/probable disordered gamblers.

C2: category 2 – non-gambler and no/low-risk disordered gamblers vs At-risk/probable disordered gamblers.

Standard errors in parentheses. ***p < 0.001, **p < 0.01, *p < 0.05.

Models 3 and 4 examine whether the effects of social strain variables on gambling disorder propensity are moderated by self-control in women. After controlling for the main effects of all predictors, only one significant interaction effect was observed for women in the category of “non-gambler.” High self-control was found to increase the impact of negative relationships with offspring on gambling severity.

**Figure 2:**
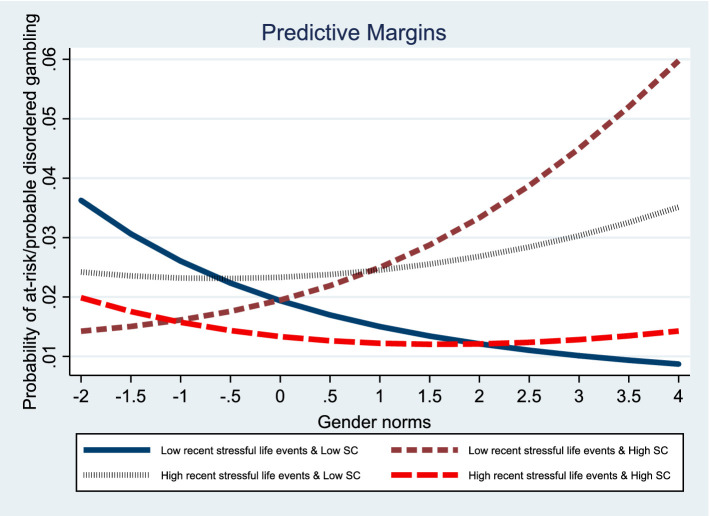
Graph of three-way interaction effects of recent stressful life events, self-control, and gender norms on gambling disorder propensity in women.

**Figure 3:**
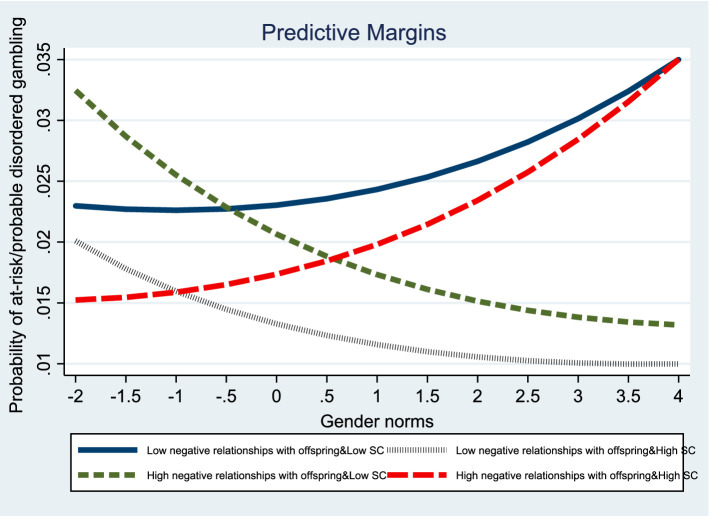
Graph of three-way interaction effects of negative relationships with offspring, self-control, and gender norms on gambling disorder propensity in women.

Models 5 and 6 further investigate the effects of the three-way interaction (social strain × self-control × gender norms) on gambling disorder propensity in women. When considering gender norms, two significant interaction effects emerged for women. Figures 2, 3 visualize the three-way interaction effects. Self-control was found to moderate the impact of recent stressful life events on the propensity for gambling disorder in women adhering to traditional gender norms. Surprisingly, self-control amplified the influence of negative relationships with offspring on the propensity for gambling disorder in women who upheld traditional gender norms.

These results highlight the crucial role of self-control in the relationship between social strain and gambling disorder, particularly when considering gender norms. They indicate the presence of gender differences as well. The self-control mitigation effect of composite strain on the propensity for gambling disorder is stronger in traditional men than in non-traditional men. In contrast, the self-control mitigation effect of recent stressful life events on the propensity for gambling disorder is stronger in traditional women than in non-traditional women. However, the self-control exacerbation effect of negative relationships with offspring on the propensity for gambling disorder is stronger in traditional women than in non-traditional women.

## 5 Discussion

This study is the first to adopt GST and self-control theory to examine the three-way interaction effects of social strain, self-control, and gender norms on the propensity for gambling disorder in Chinese married couples. To advance the GST-gendered thesis, this study investigated the roles of self-control and gender norms simultaneously in the social strain-gambling disorder nexus and found strong evidence that gender differences exist. In men, the self-control mitigation function manifests in the cumulative effects of social strain on disordered gambling likelihood when including gender norms. Specifically, the self-control mitigation effect is more pronounced in men who hold traditional gender norms than their counterparts, thus, partially supporting Hypothesis 1: the self-control mitigation effect of social strain on the propensity for gambling disorder is weaker in traditional men than in non-traditional men. While it is true that self-control reduces composite strain’s effect on the propensity for gambling disorder, the association is stronger, not weaker, for traditional men than for non-traditional men. This result challenges Gottfredson and Hirschi’s (1990) argument on the stability of self-control, which they assert is a lifetime construct that is fixed by ages 8 to 10. I believe that their result omits the influence of a social factor—marriage. Empirical evidence indicates that marriage can be a training ground for upholding self-control in men (Pronk et al., 2019; Waite and Gallagher, 2000). Studies have shown that self-control has a fluid quality, and that marriage may escalate self-control by inculcating new standards and norms, such as consideration of family members, perseverance, compromise, and motivations for self-regulation, in turn decreasing the likelihood of deviance and criminality in men. I suggest that men holding traditional gender norms are likely to view marriage as a long-term commitment. Consequently, they will exercise self-control to resist deviance in response to stress and devote more effort to maintaining a well-functioning relationship. This could explain the finding that self-control’s mitigating effect on the propensity for gambling disorder is stronger in traditional than in non-traditional men.

Conversely, in women, self-control mitigates the effects of recent stressful life events but exacerbates the effects of negative relationships with offspring on the propensity for gambling disorder; these associations are stronger for women who conform to traditional gender norms than their counterparts. The results, again, partially support Hypothesis 2: the self-control mitigation effect of social strain on the propensity for gambling disorder is stronger in traditional women than in non-traditional women. The self-control mitigation effect on the relationship between recent stressful life events and subsequent gambling disorder in traditional women supports this hypothesis. Yet, the self-control exacerbation effect on the relationship between negative relationships with offspring and the propensity for gambling disorder in traditional women runs contrary to this. The unexpected finding may be attributable to the heightened controlling practices exercised by traditional women. As mentioned earlier, self-control theory posits that women tend to exhibit higher levels of self-control than men owing to gendered parental socialization (Gottfredson and Hirschi, 1990). Such socialization is reflective of traditional gender norms, in that it more closely corrects girls’ misconduct (Jo and Bouffard, 2014; Koon-Magnin et al., 2016). Thus, traditional women with high self-control are likely to reinforce societal expectations and further exercise a controlling parenting style. Child development studies in both Eastern and Western cultures have demonstrated that controlling motherhood practices contribute to children’s poor academic and emotional functioning by undermining children’s basic need for autonomy (Barber, 1996; Cheung et al., 2016; Grolnick et al., 2002). Because of heightened controlling practices, children suffer, further inducing tension between mothers and children. Consequently, stressed mothers may become trapped in a vicious cycle of negative relationships with children and controlling parenting styles. Congruent with this argument, our findings show that the self-control exacerbation effect of negative relationships with offspring on the propensity for gambling disorder is stronger for traditional women than for non-traditional women.

The theoretical contribution of this study is that it integrates a gender perspective and establishes a foundation for empirically testing gender norms in conjunction with GST and self-control theory within a Chinese context. Through an examination of married couples, this research unveils the intricate dynamics of social strain, self-control, gender norms, and familial relationships in the realm of deviant behaviors, notably problem gambling. Within the sphere of men, the study reveals a sophisticated relationship between composite strain, self-control, and traditional gender norms, underscoring the pivotal role of marriage in fostering self-regulation. Contrary to static conceptions of self-control, the findings emphasize the fluid nature of self-regulation, highlighting marriage as a crucible for instilling values of perseverance, compromise, and familial consideration. Through the cultivation of these virtues, marriage emerges as a cornerstone for nurturing self-control and mitigating deviant behaviors among men adhering to traditional gender norms. Conversely, the research delves into the complex dynamics within traditional women, shedding light on how self-control intersects with familial relationships to influence the propensity for gambling disorder. The study elucidates how traditional gender norms can magnify the impact of self-control on gambling disorder propensity, particularly in the context of negative relationships with offspring. This unexpected result underscores the need to consider the influence of controlling motherhood practices in understanding the gendered aspects of deviant behaviors. Moving forward, future research in the domains of social strain, self-control, and gender norms should broaden its scope to encompass a diverse array of deviant behaviors, delving deeper into the complex dynamics of family structures and marital relationships. By unraveling the complexities of family dynamics and gender norms in shaping deviant behaviors, scholars can glean valuable insights into the multifaceted interplay of individual agency, societal expectations, and familial influences on the gender-deviance nexus.

The findings have practical implications for gambling treatment programs, which should address self-control and gender norms simultaneously to reduce the effects of social strain on gambling disorder. Several gambling studies show that low self-control is highly predictive of problem gambling. Therefore, it is essential for gambling treatment programs to reinforce self-control (Cheung, 2016; Hong Kong Polytechnic University, 2006). Apparently, reinforcing self-control can ameliorate social strain and disordered gambling for both male and female pathological gamblers. The prominent contribution of gender norms in conjunction with self-control for decreasing composite strain’s effect on problem gambling in traditional men, and the effect of recent stressful life events on problem gambling in traditional women, merit close attention. Therefore, gambling treatment programs should simultaneously consider the roles of self-control and gender norms in diminishing social strain and gambling disorder, especially in traditional men and women. Equally noteworthy is our discovery that the self-control exacerbation effect of negative relationships with offspring on the propensity for gambling disorder is strong in traditional women. The coercive parenting style of traditional Chinese mothers may create stress between mothers and their children. Therefore, intervention programs should also consider high self-control in conjunction with parenting style when managing negative relationships with offspring, especially in traditional Chinese mothers. Treatment programs and related research should focus on self-control, gender norms, and parenting style to reduce social strain associated with pathological gambling.

This study has limitations. First, the cross-sectional design cannot establish causal relationships or infer causality regarding bidirectional relationships in the study constructs. I found that social strain, self-control, and other control variables are precursors of gambling disorder propensity, but they can also be the consequences of pathological gambling. Further longitudinal data will provide more evidence on the causal relationships between social strain, self-control, other conditioning variables, and gambling disorder propensity across genders. Second, our data were only collected in Hong Kong, which may limit generalizability to other regions. Gender norms that associate social strain and self-control with the propensity for gambling disorder in the present study may not behave the same way for couples in other cultures. Further studies could explore whether these associations are culture-specific.

## Data Availability

The raw data supporting the conclusions of this article will be made available by the author, without undue reservation.
